# The Cpx Stress Response Regulates Turnover of Respiratory Chain Proteins at the Inner Membrane of *Escherichia coli*

**DOI:** 10.3389/fmicb.2021.732288

**Published:** 2022-01-28

**Authors:** Valeria Tsviklist, Randi L. Guest, Tracy L. Raivio

**Affiliations:** ^1^Department of Biological Sciences, University of Alberta, Edmonton, AB, Canada; ^2^Lewis Thomas Laboratory, Department of Molecular Biology, Princeton University, Princeton, NJ, United States

**Keywords:** envelope stress response, envelope biogenesis, respiration, NADH dehydrogenase I, succinate dehydrogenase, protein folding and degradation, Cpx envelope stress response

## Abstract

The Cpx envelope stress response is a major signaling pathway monitoring bacterial envelope integrity, activated both internally by excessive synthesis of membrane proteins and externally by a variety of environmental cues. The Cpx regulon is enriched with genes coding for protein folding and degrading factors, virulence determinants, and large envelope-localized complexes. Transcriptional repression of the two electron transport chain complexes, NADH dehydrogenase I and cytochrome *bo*_3_, by the Cpx pathway has been demonstrated, however, there is evidence that additional regulatory mechanisms exist. In this study, we examine the interaction between Cpx-regulated protein folding and degrading factors and the respiratory complexes NADH dehydrogenase I and succinate dehydrogenase in *Escherichia coli*. Here we show that the cellular need for Cpx-mediated stress adaptation increases when respiratory complexes are more prevalent or active, which is demonstrated by the growth defect of Cpx-deficient strains on media that requires a functional electron transport chain. Interestingly, deletion of several Cpx-regulated proteolytic factors and chaperones results in similar growth-deficient phenotypes. Furthermore, we find that the stability of the NADH dehydrogenase I protein complex is lower in cells with a functional Cpx response, while in its absence, protein turnover is impaired. Finally, we demonstrated that the succinate dehydrogenase complex has reduced activity in *E*. *coli* lacking the Cpx pathway. Our results suggest that the Cpx two-component system serves as a sentry of inner membrane protein biogenesis, ensuring the function of large envelope protein complexes and maintaining the cellular energy status of the cell.

## Introduction

Gram-negative bacteria possess a unique cell envelope structure consisting of three principal layers: the outer membrane (OM), the peptidoglycan cell wall and the inner membrane (IM) (Ruiz et al., 2006; Silhavy et al., 2010). This complex multicomponent structure functions as a protective barrier, communicates changes in the external environment, and maintains the shape, stability, and rigidity of the cell (Ruiz et al., 2006; Holst et al., 2010; Silhavy et al., 2010). In addition, the bacterial envelope ensures successful host infection and colonization due to a multitude of envelope-localized virulence factors, together with appropriate metabolism and growth of the cell (Hews et al., 2019). Bacteria have evolved a sophisticated regulatory network to maintain envelope homeostasis. Under conditions where the process of membrane biogenesis and hence envelope homeostasis is impaired, bacterial cells activate several stress response systems, including the CpxAR pathway.

CpxAR is a canonical two-component signal transduction system (TCS) that consists of the membrane-localized sensor histidine kinase CpxA and cytoplasmic response regulator CpxR (Grabowicz and Silhavy, 2017a; Delhaye et al., 2019a; Hews et al., 2019). Under non-inducing conditions, the phosphatase activity of CpxA maintains CpxR in a dephosphorylated or inactive state. When an inducing cue is present, CpxA autophosphorylates at a conserved histidine residue and transfers a phosphate group to CpxR (Raivio and Silhavy, 1997). The downstream targets of CpxR transcriptional regulation include genes whose products are involved, either directly or indirectly, in protein folding and degradation in the bacterial envelope (Pogliano et al., 1997; Raivio and Silhavy, 2001). The Cpx regulon is enriched with genes encoding virulence factors, small regulatory RNAs (sRNAs), multidrug efflux systems and peptidoglycan modifying factors (Raivio, 2014; Hews et al., 2019). Two auxiliary proteins involved in the Cpx regulatory pathway are the periplasmic protein CpxP, one of the most highly expressed members of the Cpx regulon, proposed to inhibit activation of CpxA, and the OM lipoprotein NlpE, which is thought to activate CpxA upon surface adhesion and to sense stresses related to defects in lipoprotein trafficking (Danese et al., 1995; Danese and Silhavy, 1998; Raivio et al., 1999; Otto and Silhavy, 2002; Grabowicz and Silhavy, 2017b; Delhaye et al., 2019b).

The Cpx system is triggered by a variety of signals, including alkaline pH, aminoglycoside antibiotics, NlpE overexpression, aberrant expression of Pap pilus subunits, and mutation of the IM protease FtsH (Danese et al., 1995; Nakayama and Watanabe, 1995; Jones et al., 1997; Mileykovskaya and Dowhan, 1997; Danese and Silhavy, 1998; Hung et al., 2001; Shimohata et al., 2002; Kohanski et al., 2008). These activating cues are related to potentially lethal accumulation of misfolded or mislocalized proteins at the bacterial envelope. To restore the integrity of the envelope, the Cpx response upregulates proteolytic factors and periplasmic protein folding factors, including the disulfide bond oxidoreductase DsbA, the peptidyl-prolyl isomerase PpiA, the chaperones Spy and CpxP (Danese and Silhavy, 1997, 1998; Pogliano et al., 1997; Raivio et al., 2000), and the protease/chaperone DegP (Cosma et al., 1995; Danese and Silhavy, 1997). For instance, non-productive assembly of P pili results in the activation of the Cpx pathway and subsequent upregulation of DegP, which degrades misfolded pilin subunits (Hung et al., 2001). Additionally, the Cpx pathway directly represses transcription of high molecular weight protein complexes as an adaptation to stresses that lead to protein misfolding (Raivio et al., 2013; Raivio, 2014; Hews et al., 2019). The electron transport chain (ETC) incorporates some of the largest multiprotein complexes in the *Escherichia coli* IM, making them a primary target of Cpx regulation (Guest et al., 2017).

The majority of ATP in the cell is produced via oxidative phosphorylation during aerobic growth. The ETC complexes convert the energy of reducing equivalents, such as NADH or FADH_2_, into a proton electrochemical gradient across the membrane. This electrochemical gradient drives ATP synthesis via ATP synthase and for other energy consuming processes in the cell, including active transport and flagellar motion (Yoshida et al., 2001; Kaila et al., 2010; Price and Driessen, 2010; Borisov et al., 2011; Kaila and Wikström, 2021). The respiratory chain is composed of primary dehydrogenases and terminal oxidases coupled by the quinone pool. The first steps of electron transport are catalyzed by NADH dehydrogenase (NDH-I), the largest complex of the ETC, and the entry point for electrons carried by NADH (Brandt, 2006; Kerscher et al., 2008). In *E*. *coli*, the *nuo* operon contains 13 genes, *nuoA-N*, where *nuoC* encodes a fused version of NuoC and NuoD subunits (Braun et al., 1998; Price and Driessen, 2010; Friedrich et al., 2016). NDH-I is an L-shaped multisubunit structure composed of a hydrophobic membrane arm, protruding into the lipid bilayer, and a hydrophilic peripheral arm that extends into the cytoplasm (Hofhaus et al., 1991; Guénebaut et al., 1998; Baranova et al., 2007). The processes of proton translocation and quinone binding take place in the membrane arm consisting of NuoA, H, and J-N, whereas NADH oxidation is the function of the peripheral arm comprising NuoB, CD, E, F, G, and I subunits (Hofhaus et al., 1991).

Succinate dehydrogenase (SDH) is a unique membrane-bound enzyme that is a common component of the ETC and the tricarboxylic acid (TCA) cycle (Lestienne and Desnuelle, 1999). Within the TCA cycle, SDH oxidizes succinate to fumarate sequestering two electrons that are then used for the reduction of ubiquinone in the membrane (Yankovskaya et al., 2003). It is composed of four non-identical subunits encoded by the *sdhCDAB* operon, where SdhA and SdhB are the cytoplasmic catalytic subunits which contain the flavin adenine dinucleotide and iron-sulfur (Fe-S) cluster cofactors, respectively. SdhC and SdhD compose the membrane-integral part of the enzyme and contain the ubiquinone binding site and the heme b cofactor (Yankovskaya et al., 2003; Tran et al., 2006; Price and Driessen, 2010). The absence of either SdhC or SdhD structural components leads to unstable SDH activity in the cytoplasm and perturbs ubiquinone reduction at the IM (Nakamura et al., 1996).

The Cpx-mediated downregulation of *nuo* (NDH-I) and *cyo* (cytochrome *bo3*) operons, and its effect on aerobic respiration were recently demonstrated in enteropathogenic *E*. *coli* (EPEC), which aligns with previous RNA transcriptome findings (Raivio et al., 2013; Guest et al., 2017; Dbeibo et al., 2018). These studies proposed that, in the presence of envelope stress, *de novo* synthesis of these complexes is repressed to reduce protein traffic within the IM, and maintenance of existing complexes is performed via upregulation of the Cpx-regulated protein folding and degrading factors (Guest et al., 2017). While transcriptional repression of the genes encoding NDH-I and cytochrome *bo*_3_ complexes of ETC has been described, additional levels of regulation remain to be investigated.

In this study, we show that the Cpx pathway regulates the expression of the SDH enzyme transcriptionally and provide evidence that Cpx-dependent regulation extends beyond transcriptional repression of the ETC complexes. We describe the Cpx-dependent regulation of protein turnover of the NuoA subunit of the NDH-I complex and identify several Cpx-regulated protein folding and degrading factors associated with this regulation. Intriguingly, our data suggest that in the absence of the Cpx pathway the function of the SDH complex is impaired, which suggests that the Cpx response plays an essential role in maintaining the energy status of the cell, ensuring the stability, activity, and proper turnover of the ETC complexes.

## Materials and Methods

### Bacterial Strains and Growth Conditions

All bacterial strains and plasmids used in this study are listed in Supplementary Table 1. Cultures were grown and maintained in LB broth or M9 minimal medium (Difco) at 37°C with shaking at 225 rpm, with the exception of strains bearing the *cpxA24* mutation, which were grown at 30°C in the presence of amikacin (3 μg/ml) to prevent accumulation of suppressors as previously described (Raivio et al., 1999). Isopropyl-β-D-thiogalactopyranoside (IPTG, Invitrogen) was added to a concentration of 0.1 mM to induce gene expression from pCA24N- and pMPM-K3-based vectors. Antibiotics (Sigma) were added as necessary at the following concentrations: chloramphenicol (Cam), 25 μg/mL; kanamycin (Kan), 30 or 50 μg/mL; ampicillin (Amp), 100 μg/mL; spectinomycin (Spc), 25 μg/ml.

### Strain and Plasmid Construction

All BW25113 mutants were taken from the Keio collection (Baba et al., 2006). An MC4100 Δ*cpxR* knockout mutant was generated using P1 transduction to move the desired mutant allele from the Keio collection (Baba et al., 2006) into wildtype MC4100 as previously described (Silhavy et al., 1984). The inducible pCA24N-based plasmids used in this study were obtained from the ASKA collection (Kitagawa et al., 2005). Transcriptional luminescent reporters containing the promoter regions of *cpxP* and *nuoA* were constructed as previously described (Price and Raivio, 2009; Wong et al., 2013; Guest et al., 2017). The p*sdhC*::*lux* reporter was constructed similarly. Briefly, the promoter region of *sdhC* gene was amplified by PCR, using PsdhCFwdCln and PsdhCRevCln primers (Supplementary Table 2). Purified PCR products and the pJW15 vector (MacRitchie et al., 2008) were digested with *Eco*RI and *Bam*HI (Invitrogen), and the insert was ligated upstream of the *luxABCDE* operon in the pJW15 plasmid. Correct insertion of the promoter sequence was verified by PCR and sequencing using pNLP10F and pNLP10R primers (Supplementary Table 2).

For the construction of pTrc-*nlpE* vector, *nlpE* was amplified via PCR with recombinant Taq polymerase (Invitrogen) using nlpE_NcoI_F and nlpE_WT_*Hin*dIII_R primers (Supplementary Table 2). PCR products were purified using a QIAGEN QIAQuick PCR purification kit according to the manufacturers protocol. Amplified *nlpE* was digested along with purified pTrc99A using Fast Digest *Eco*RI and *Hin*dIII (Thermo Scientific). Digests were purified using the aforementioned PCR purification kit and were then ligated together using T4 DNA ligase. Ligations were then transformed into One Shot TOP10 chemically competent cells (Thermo Fisher). Correct insertion of the *nlpE* sequence was verified by PCR and sequencing using pTrc99A_F and pTrc99A_R primers (Supplementary Table 2).

The pMPM-NuoA-3 × FLAG plasmid was constructed by amplifying *nuoA* from the E2348/69 chromosome via PCR using primers nuoAFLAGFwd and nuoAFLAGrev (Supplementary Table 2). Primer nuoAFLAGrev contains the nucleotide sequence to insert a triple FLAG-tag directly upstream of the *nuoA* stop codon. PCR was performed using high-fidelity phusion DNA polymerase (ThermoFisher) according to the manufacturers protocol with the addition of 10% betaine. The DNA band corresponding to *nuoA*-3 × FLAG DNA was gel extracted and cleaned using the GeneJet gel purification kit (Fermentas). Both *nuoA*-3 × FLAG and pMPM-K3 DNA were digested with the *Hin*dIII and *Xba*I restriction endonucleases (Invitrogen) according to the manufacturers protocol. *nuoA*-3 × FLAG DNA was then ligated downstream of an IPTG inducible P*lac* promoter in the pMPM-K3 vector and transformed into One Shot TOP10 chemically competent *E*. *coli* (Invitrogen) as per the manufacturer’s protocol. PCR and DNA sequencing were used to confirm the presence of *nuoA*-3 × FLAG fragment within pMPM-K3 using M13F and M13R primers (Supplementary Table 2). All DNA sequencing was performed by The University of Alberta Molecular Biology Services Unit (MBSU). All plasmids in this study were transformed into *E*. *coli* strains via chemical competency (Silhavy et al., 1984).

### Luminescence Assay

Strains containing p*cpxP*::*lux*, p*sdhC*::*lux*, or p*nuoA*::*lux* reporter plasmids were grown overnight in LB broth with shaking at 37°C. Cells were pelleted by centrifugation and washed twice with phosphate-buffered saline (PBS). The cell density was standardized to OD_600_ 1.0, pelleted, and resuspended in 1 mL of 1 × PBS. Standardized cultures were serially diluted 10-fold and 10 μL of each dilution was spotted onto M9 minimal agar containing 0.4% glucose, malic acid, or succinic acid (Sigma). Glucose-succinate gradient agar plates were created by pouring M9 containing 0.4% glucose into an angled plate. The layer was allowed to solidify in the inclined position and the second portion of agar containing 0.4% succinic acid was poured into the plate, placed on a level surface and allowed to solidify (Weinberg, 1959). Agar pH was adjusted to 7.0 with sodium hydroxide. Bacteria were grown for 24–48 h at 37°C statically. Luminescence was determined by imaging the light produced by strains using the UVP Colony Doc-It Imaging Station (Biorad). Luminescence was quantified using Fiji (ImageJ).

For the assay performed in liquid medium, bacteria were grown overnight as described above, subcultured at a dilution factor of 1:100 into 5 ml of fresh LB broth and incubated for 2 h at 37°C with aeration. 200 μL of each subculture was aliquoted into a black-bottomed 96-well plate, and luminescence in counts per second (CPS) and OD_600_ were measured every 1 h for 7 h post-subculture using a PerkinElmer VICTOR™ X3 multilabel reader. Luminescence and OD_600_ values measured from a blank well containing uncultured LB were subtracted from each sample. CPS was standardized to the OD_600_ to correct for differences in cell numbers between samples. All experiments contained three technical replicates and were carried out three times.

### Western Blot Analysis

Samples used for Western blot analysis were prepared by diluting overnight cultures 1:100 into 25 ml fresh LB containing appropriate concentrations of antibiotics. Bacteria were grown at 37°C with shaking to an OD_600_ of ∼ 0.35. IPTG was added to a concentration of 0.1 mM and bacteria were grown for an additional 30 mins as before. 2 × 1 mL samples were collected. Cells were pelleted by centrifugation at 21,130 × *g* for 1 min. One sample was resuspended in 50 μL 2 × Laemmli sample buffer (Sigma) and the other sample was resuspended in 50 μL 1 × PBS. Protein concentration was determined from the sample resuspended in phosphate-buffered saline using the Pierce BCA protein assay kit (ThermoFisher) according to the manufacturers’ protocol. Samples resuspended in 50 μL 2 × Laemmli sample buffer were denatured by boiling for 5 mins. Sample volumes were standardized according to their determined protein concentrations and separated on a 12% SDS gel at 110 V for 1.5 h in Tris–glycine running buffer (10% SDS, 250 mM Tris, 1.2 M glycine).

Proteins were transferred onto a nitrocellulose membrane via the trans-blot semi-dry transfer system (Bio-Rad) at 15 V for 22 mins using semi-dry Towbin transfer buffer (78 mM glycine, 1.3 mM SDS, 20% methanol). Membranes were blocked in 5% MTS (2.5% skim milk powder, 154 mM NaCl, 1 mM Tris) or 2% BSA (154 mM NaCl, 1 mM Tris, 1% Tween 20, 2% bovine serum albumin) for 1 h at room temperature with shaking at 10 rpm. Primary α-FLAG (Sigma, BioLegend), α-PhoA (Abcam) and α-RNAPα (BioLegend) antibodies were diluted by a factor of 1:5,000 into 5% MTS or 2% BSA. Membranes were incubated with the primary antibody for 1 h at either room temperature with shaking at approximately 10 rpm or overnight at 4°C with rocking. Following incubation with the primary antibody, membranes were washed for 5 mins in wash solution (154 mM NaCl, 1 mM Tris, 1% Tween 20) four times. Alkaline-phosphatase (AP) anti-rabbit secondary antibodies (Sigma) were diluted at a factor of 1:10,000 in 5% MTS and were used to detect the α-PhoA (Abcam) and α-FLAG (Sigma) primary antibodies. IRDye®680RD Goat anti-Mouse 925-68070 and IRDye®800CW Goat anti-Rabbit 925-32211 secondary antibodies (LI-COR) were diluted at a factor of 1:15,000 in 5% MTS and were used to detect the α-RNAPα (BioLegend) and α-FLAG (BioLegend) primary antibodies, respectively.

Membranes were then incubated with the secondary antibody for 1 h at room temperature with shaking at approximately 10 rpm. Membranes were washed following incubation with the secondary antibody as before. Proteins from the membranes incubated with AP secondary antibodies were detected using the Immun-Star alkaline phosphatase chemiluminescence kit (Bio-Rad). All membranes were imaged with the Bio-Rad ChemiDoc MP imaging system. Quantification of each band compared to the wildtype was performed using band intensity analysis in Fiji (ImageJ). Experiments were performed in biological triplicates, and a representative blot is shown in each case.

### Protein Stability Assay

Bacteria were grown overnight in 5 mL LB at 37°C with shaking. The following day, bacteria were subcultured at a 1:100 dilution into 25 mL fresh LB and grown at 37°C with shaking to an OD_600_ ∼ 0.5. IPTG was then added to a final concentration of 0.1 mM and bacteria were grown to an OD_600_ of 1.0. 1 mL samples were collected and cells were pelleted by centrifugation at 21,130 × *g* for 1 min. After the supernatant was removed, cells were resuspended in 50 μL 2 × Laemmli sample buffer (Sigma). Immediately after the sample was removed, the protein synthesis inhibitor chloramphenicol was added to the remaining culture at a concentration of 100 μg/mL. The culture was incubated at 37°C and shaken at 225 rpm. 1 mL of culture was collected right before the addition of chloramphenicol and at 1, 5, 10, 20, 30, 45, 90, and 120 min(s) after the addition of chloramphenicol. Sample collection at each timepoint was performed as described above. 10 μL of each sample was loaded onto a 12% SDS polyacrylamide gel. Incubation with primary and secondary antibodies, detection and band quantification were performed as described above. Each experiment was repeated three times, and a representative blot is shown in each case.

### Succinate Dehydrogenase Activity Assay

Succinate dehydrogenase activity was measured using a kit (Abcam). Samples were prepared following the manufacturer’s protocol by diluting overnight cultures 1:50 into 5 mL fresh LB and growing them to OD_600_ ∼0.5 at 37°C with shaking. 1 mL of culture was taken, pelleted by centrifugation, washed with 1 × PBS and pelleted again. Cultures were then standardized to the same optical density OD_600_ of 1.0 in the final volume of 1 mL by adding an appropriate volume of 1 × PBS, pelleting, and resuspending in 200 mL of ice-cold SDH Assay Buffer (Abcam). Cell lysis was performed by sonication. Samples were centrifuged at 10,000 × *g* for 5 mins and the supernatant was transferred into a fresh tube. 50 μL of each sample was loaded into a 96-well plate, and 50 μL of SDH reaction mix containing the SDH substrate mix, and the probe (Abcam) was added to them. Absorbance at 600 nm was measured for 30 mins at 25°C in kinetic mode using the Cytation5™ Cell Imaging Multi-Mode Reader (BioTek). The succinate dehydrogenase activity of the samples was calculated according to the manufacturers’ directions. Data is representative of the means and standard deviations of three biological replicates.

### Minimal Media Growth Assays

Wildtype BW25113 or knockout mutants were grown overnight in 2 mL LB at 37°C with shaking. The following day, cells were pelleted by centrifugation and washed twice with 1 × PBS. The density was standardized to OD_600_ 1.0 by suspending an appropriate volume of cells in 1 ml of 1 × PBS. 20 μL of each sample was loaded into a 96-well plate, each well containing 180 μL of either 0.4% glucose (Sigma), 0.4% malic acid (Sigma) or 0.4% succinic acid (Sigma) M9 minimal medium (Difco), pH 7.0. The plate was incubated in the Cytation5™ Cell Imaging Multi-Mode Reader with 330 rpm shaking at 37°C for 48 h.

## Results

### Regulation of the Succinate Dehydrogenase Complex by the Cpx Response

Recent studies have demonstrated that the Cpx envelope stress response regulates the expression and activity of NDH-I and cytochrome *bo*_3_ complexes in EPEC (Guest et al., 2017). In addition, Cpx-dependent downregulation of genes encoding the succinate dehydrogenase complex was observed in a large microarray dataset; however, the exact mechanism of regulation has not been revealed (Raivio et al., 2013). To investigate the impact of the Cpx pathway on the SDH, we grew strains carrying either the vector control or a reporter plasmid in which 500 bp upstream of the *sdhCDAB* operon were fused to the *luxCDABE* gene cluster in liquid LB medium aerobically for 24 h. Luminescence activity of the p*sdhC*::*lux* reporter in the presence or absence of the Cpx pathway was measured every hour until the stationary phase was reached. We chose to use the luminescence activity produced by the p*cpxP*::*lux* reporter gene as a positive control, since CpxP is one of the most upregulated members of the Cpx regulon (Price and Raivio, 2009).

**FIGURE 1:**
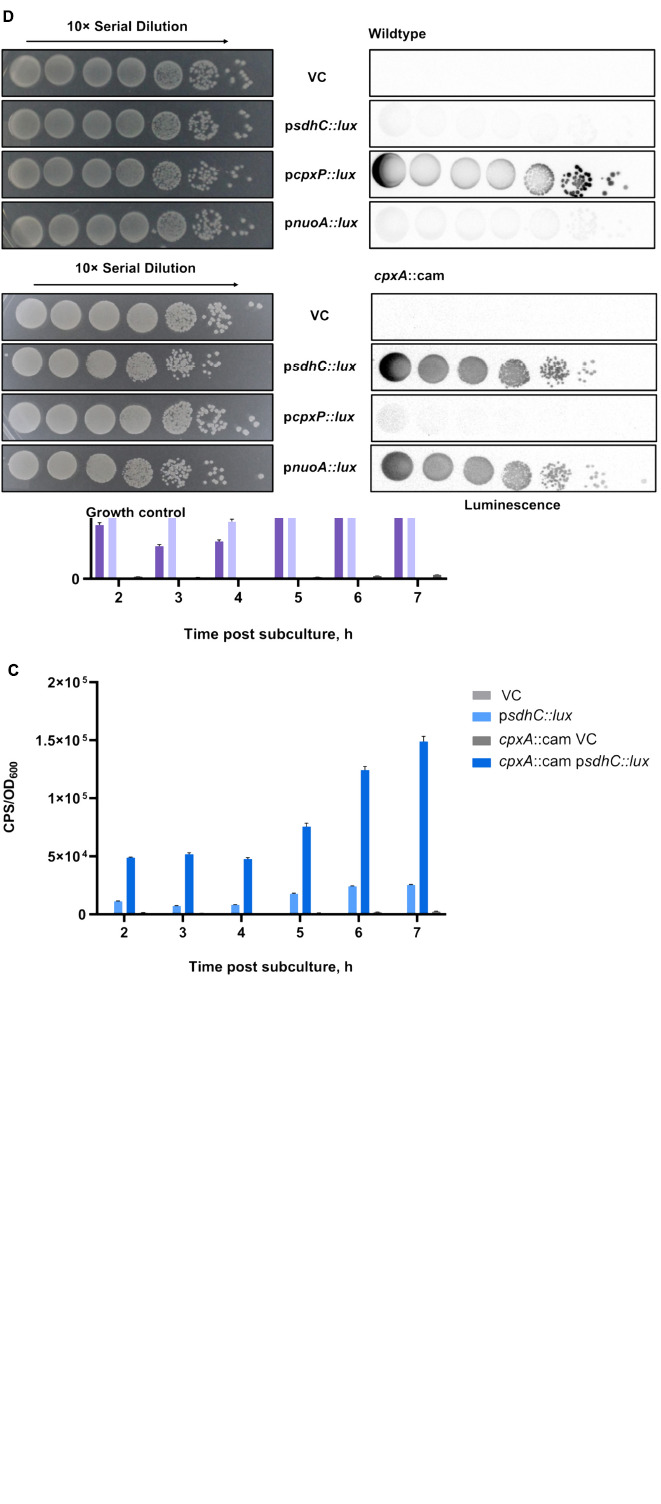
Transcription of the *sdh* operon encoding the succinate dehydrogenase complex is downregulated by the Cpx stress response. Overnight cultures of wildtype *Escherichia coli* MC4100 or a *cpxA*::cam mutant harboring either pJW15 (vector control) or the **(A)** p*cpxP*::*lux*, **(B)** p*nuoA*::*lux*, or **(C)** p*sdhC*::*lux* reporter plasmids were subcultured 1:100 into fresh LB medium and incubated at 37°C with shaking. The luminescence and the OD_600_ were measured every hour for the duration of 7 h and a final measurement was taken at 24 h. Data correspond to the mean values from three biological replicates. Error bars depict standard deviations (SDs). **(D)** Luminescence activities are shown for wildtype *E*. *coli* MC4100 or a *cpxA*::cam mutant harboring either pJW15 (vector control) or the p*nuoA*::*lux*, p*sdhC*::*lux*, or p*cpxP*::*lux* reporter plasmids. Luminescence was determined by imaging the luminescence of the strains after 24 h of growth on LB plates.

In agreement with previous observations, the expression of the p*cpxP*::*lux* reporter in the wildtype BW25113 was higher than in a *cpxA* null background at every stage of growth (Figure 1A). Along with *cpxP*, we assayed the activity of the p*nuoA*::*lux* reporter and validated its Cpx-dependent downregulation as reported in the past (Figure 1B; Guest et al., 2017). Loss of Cpx response resulted in a ∼2-fold increase in the p*nuoA*::*lux* activity compared to that of the wildtype. When we assayed the luminescence activity of the p*sdhC*::*lux* reporter, we found similar increases in the absence of a functional Cpx response, while the wildtype activity of the Cpx TCS lead to transcriptional repression of the *sdhC* promoter (Figure 1C).

To corroborate the luminescence profile of the control and ETC reporters in liquid LB medium, we also examined expression of the reporter genes during growth on solid LB medium (Figure 1D). The patterns of p*nuo*::*lux*, p*sdhC*::*lux* and p*cpxP*::*lux* reporter activity observed on solid medium resembled those in the liquid medium assay. In the wildtype strain, the p*cpxP*::*lux* reporter was strongly activated, while light was not detectable in a *cpxA* null mutant background. This pattern was reversed for the p*nuo*::*lux* and p*sdhC*::*lux* reporters which demonstrated low activity in the presence of the intact Cpx pathway (Figure 1D).

Our results suggest that the Cpx TCS could directly inhibit the expression from the *sdhC* promoter. We thus decided to examine the region upstream of *sdhCDAB* for the presence of any putative CpxR binding sites and identified one approximately 150 bp downstream of the predicted *sdhC* transcription start site (TSS) by using Virtual Footprint^1^ (Münch et al., 2005; Supplementary Figure 1).

### Succinate Dehydrogenase Activity Is Affected by Excessive Activation or Absence of the Cpx Response

**FIGURE 2:**
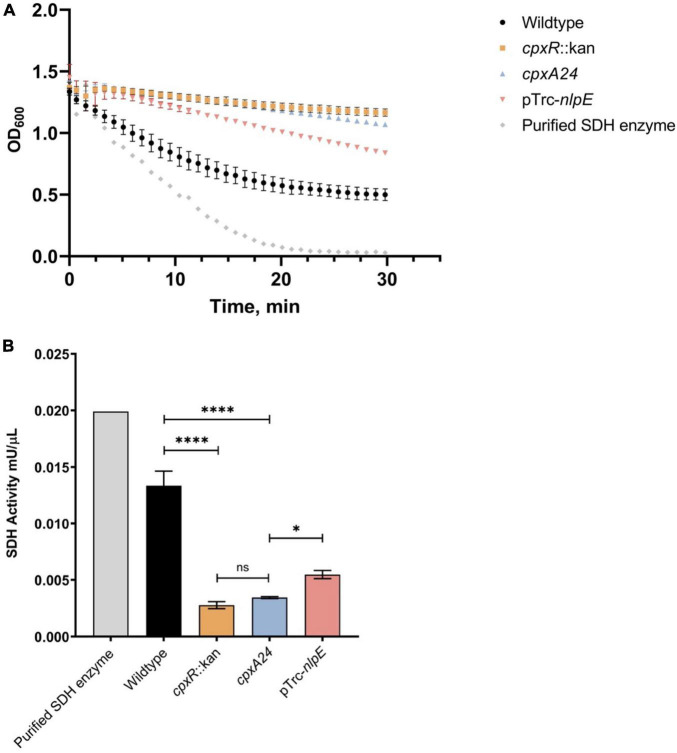
Succinate dehydrogenase (SDH) activity is reduced by excessive activation or absence of the functional Cpx response. Wildtype MC4100, *cpxR*::kan, *cpxA24* and MC4100 harboring NlpE overexpression plasmid (pTrc-*nlpE*) were subcultured from their overnight cultures 1:50 into 5 mL fresh LB and grown to OD_600_ ∼0.5 at 37°C with shaking. 1 mL samples, standardized to the same optical density OD_600_, were pelleted, and cell pellets were resuspended in 200 μL of ice-cold SDH Assay Buffer (Abcam). Samples were prepared as per manufacturers’ protocol, loaded into a 96-well plate, and mixed with 50 μL of SDH reaction mix (Abcam). **(A)** Assay data showing reduction of the DCIP artificial electron acceptor, accompanied by the color change of the dye from blue to colorless (A_600_). Absorbance at 600 nm was measured every minute for 30 mins at 25°C with shaking using the Cytation5™ Cell Imaging Multi-Mode Reader (BioTek). **(B)** Succinate dehydrogenase activity of each sample was calculated as per manufacturers’ protocol and plotted. All data correspond to the means and standard deviations of three replicate cultures. Asterisks indicate a statistically significant difference from the relevant wildtype control [****P ≤ 0.0001 (one-way ANOVA with Tukey’s *post hoc* test)]. NS indicates no statistically significant difference in SDH activity.

Our experimental results with the p*sdhC*::*lux* reporter predict that the enzymatic activity of SDH will be lower in the presence of an active Cpx TCS relative to a mutant lacking the Cpx response. To test this hypothesis, we assayed the rates of succinate oxidation in the wildtype *E*. *coli* strain, as well as in *cpxR*::kan and *cpxA24* mutants, reflecting a deactivated and constitutively activated Cpx pathway, respectively (Raivio and Silhavy, 1997). We also included a strain overexpressing NlpE as an alternative way to induce the Cpx response (Figure 2). In this assay, the oxidation of succinate is accompanied by the transfer of electrons to an artificial electron acceptor (probe), which changes color depending on the enzymatic activity of the sample. When in the oxidized state, the probe is blue with maximal absorption at 600 nm, whereas when reduced it becomes colorless. We found that the activity of the SDH complex decreased upon activation of the Cpx system in both the *cpxA24* mutant and the NlpE overexpression backgrounds. This result was predictable based on the repressive effect of the Cpx response on the *sdhC* promoter observed in Figures 1C,D.

Surprisingly, the loss of the Cpx response did not result in higher SDH activity, as might be anticipated with the relieved inhibition of *sdhCDAB* transcription. Instead, knocking out *cpxR* resulted in decreased succinate oxidation rates, equivalent to those observed in the presence of the strongly Cpx-activating *cpxA24* allele (Figure 2B). This result is reminiscent of a previously observed decrease in the oxygen consumption rate in the Δ*cpxR* mutant, despite the fact that transcription of the operons encoding NDH-I and cytochrome *bo*_3_ are upregulated in this background (Guest et al., 2017). Together, these findings suggest that the Cpx TCS not only transcriptionally regulates the SDH protein complex, but also regulates factors responsible for its proper activity and biogenesis.

### The Cellular Need for the Cpx Response Increases When Respiratory Complexes Are More Prevalent or Active

**FIGURE 3:**
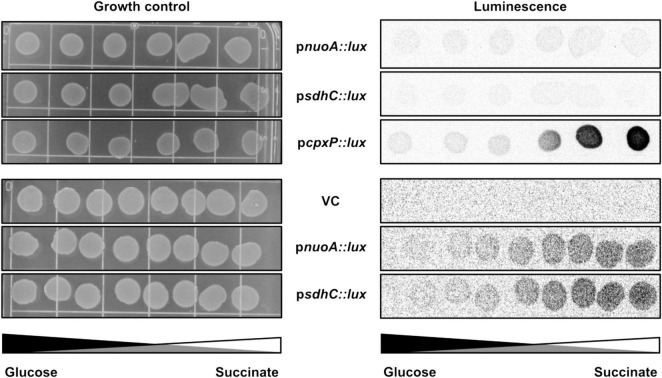
Demand for aerobic respiration due to the presence of non-fermentable carbon sources induces the Cpx pathway. Luminescence activities are shown for wildtype *E*. *coli* MC4100 harboring either pJW15 (vector control) or the p*nuoA*::*lux*, p*sdhC*::*lux*, or p*cpxP*::*lux* reporter plasmids. Luminescence of the strains growing on M9 minimal media plates containing a gradient of 0.4% glucose and 0.4% succinate, pH 7.0 was determined by imaging the luminescence of the spots after 48 h of incubation at 37°C.

*Escherichia coli* is capable of growing on a number of different sugar substrates, generating the majority of its ATP via the process of oxidative phosphorylation (Ammar et al., 2018). Carbon sources that cannot be utilized through the process of substrate-level phosphorylation, or fermentation, are called non-fermentable and require a functional ETC for sufficient energy generation. Such carbon sources include succinate, malate and glutamate (Prüss et al., 1994; Chang et al., 2004; McNeil et al., 2012). Poor growth of NDH-I mutants on either malate or succinate has been proposed to reflect low energy conservation efficiency due to a low level of ATP inside the cells (Kervinen et al., 2004). Previously reported data and our observations suggest that the Cpx TCS affects transcription and function of membrane complexes required for growth on non-fermentable carbon sources, including those associated with electron transport, the TCA cycle and oxidative phosphorylation (Figures 1B–D; Raivio et al., 2013; Guest et al., 2017). We thus hypothesized that the activity of the Cpx pathway would be required under conditions that create increased demand for respiration To test this, we spotted strains bearing luminescent reporter plasmids containing p*nuoA*::*lux*, p*sdhC*::*lux*, or p*cpxP*::*lux* promoter fusions along a glucose-succinate carbon source concentration gradient on minimal medium (Figure 3). We found that expression of the *cpxP* promoter increases as the concentration of succinate increases, whereas in the presence of glucose p*cpxP*::*lux* demonstrates background levels of luminescence comparable to the empty vector control. None of the reporters were activated to high levels in the presence of glucose, which supports our hypothesis that growth under conditions that increase demand for respiration leads to Cpx pathway activation. Due to high p*cpxP*::*lux* activity, *nuoA* and *sdhC* promoter activities were not detected by the imaging system on short exposure times, therefore we spotted the strains containing ETC reporters on a different plate and assayed them separately to image the lower luminescence activity (Figure 3). As expected, activity of the p*nuoA*::*lux* and p*sdhC*::*lux* reporters increased commensurate with the succinate concentration gradient. Notably, the expression of these transcriptional reporters was much higher in the absence of the Cpx response, which supports our previous findings in Figures 1B–D (data not shown). Based on the results of this experiment, we suggest that the cellular need for Cpx-mediated stress adaptation increases when respiratory (and possibly other) protein complexes are in increased demand.

### The Cpx Response Regulates NuoA Protein Levels

The Cpx response directly represses *nuo* operon transcription to ensure envelope integrity during stress and reduce excessive protein traffic within the IM (Guest et al., 2017; Figure 1B). The possibility of additional levels of regulation has been hypothesized because the oxygen consumption in EPEC was found to be reduced in cells with either an active or inactive Cpx pathway (Guest et al., 2017). To determine whether the Cpx response regulates respiratory proteins beyond transcription, we constructed a plasmid that expresses a triple FLAG-tagged NuoA subunit of the NDH-I complex from an exogenous, IPTG inducible promoter. We chose NuoA protein due to its scaffolding role in the assembly of the NDH-I membrane arm (Friedrich et al., 2016). Given that the NuoA-3 × FLAG construct is expressed from a CpxR-independent IPTG-inducible promoter, any effects of Cpx pathway on NuoA protein levels should be independent of transcription. To confirm that the NuoA-3 × FLAG fusion protein is functional, we complemented a chromosomal Δ*nuoA* mutant that has a growth defect on minimal medium supplemented with malate, with either the empty vector pMPM-K3 or the plasmid carrying NuoA-3 × FLAG. The exogenously expressed NuoA restored growth of the Δ*nuoA* mutant, demonstrating that the protein was expressed properly and was functional, whereas complementation with an empty vector resulted in the absence of growth (Supplementary Figure 2). All strains demonstrated similar growth levels on minimal medium supplemented with glucose, which is a fermentable carbon source.

**FIGURE 4:**
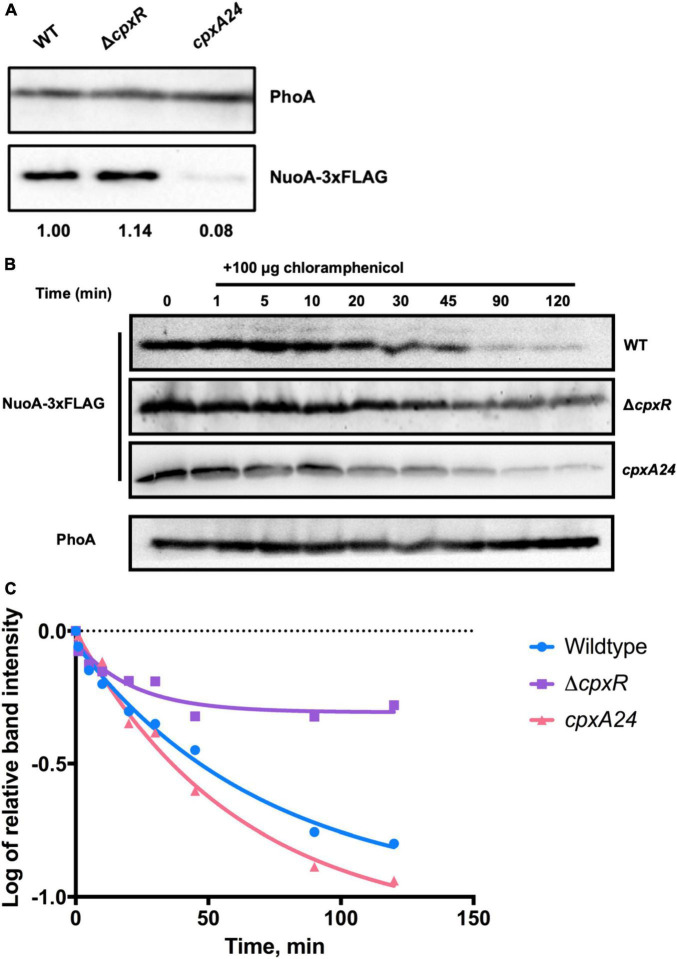
The Cpx response affects NuoA-3 × FLAG protein levels. Western blots utilizing anti-FLAG tag antibody were performed on wildtype EPEC, *cpxA24* or Δ*cpxR* mutants **(A)** or wildtype BW25113, *cpxA24* or Δ*cpxR* mutants **(B,C)** containing the pMPM-*nuoA*-3 × FLAG expression vector **(A)** at a single time-point, where band intensity is represented by the value relative to the wildtype NuoA-3×FLAG protein level and **(B)** over time after 100 μg of chloramphenicol was added to inhibit protein synthesis. Samples were taken at 0 (before addition) 1, 5, 10, 20, 30, 45, 90, and 120 min after addition to assay NuoA-3 × FLAG protein degradation. PhoA protein levels served as a loading control. Proteins were detected using the Immun-Star alkaline phosphatase chemiluminescence kit (Bio-Rad) and the Bio-Rad ChemiDoc MP imaging system. **(C)** Relative band quantification was performed using band intensity analysis in ImageJ. The protein stability assay data were fit with a one-phase exponential decay curve to determine protein half-life using the Prism v7.0c (GraphPad) software.

We assayed the amount of NuoA-3 × FLAG protein in wildtype, Δ*cpxR* and *cpxA24* strains of *E*. *coli* via Western blotting and observed a decrease in NuoA-3 × FLAG levels when the Cpx response was constitutively activated. A slight accumulation of the protein was detected in the absence of Cpx response (Figure 4A). Expression of NuoA-3 × FLAG in the *cpxA24* mutant was 8% that of the wildtype strain 30 mins after induction, whereas NuoA-3 × FLAG levels were increased by 14% in the Δ*cpxR* mutant compared to the wildtype (Figure 4A).

To gain more insight into how the activity of the Cpx response affects protein turnover over time, we performed a protein stability assay by utilizing the protein synthesis inhibitor chloramphenicol and monitoring the rate of NuoA-3 × FLAG degradation over 120 mins after translation has been halted (Figure 4B). Relative quantification of the bands showed that in the absence of the Cpx response, approximately half of the starting amount of NuoA-3 × FLAG protein was degraded in 45 mins, which was 25 mins longer than in the wildtype strain, indicating slower protein turnover (Figure 4C and Supplementary Table 3). Unexpectedly, the rates of NuoA-3 × FLAG degradation over the course of the experiment were comparable between the *cpxA24* and the wildtype strains, although the total amount of protein degraded by the end of the experiment was larger in the *cpxA24* (Figures 4B,C and Supplementary Table 3). Together, our results suggest that Cpx-regulated protein degrading factors are responsible for faster and more efficient turnover of NuoA-3 × FLAG proteins in the wildtype strain. Counterintuitively, an increased rate of degradation does not seem to be the only reason for the diminished amount of NuoA observed in *cpxA24* strain backgrounds (Figure 4A).

### Cpx-Regulated Protein Folding and Degrading Factors Affect Growth During High Respiratory Demand

We found that the rate of NuoA-3 × FLAG proteolysis was reduced when the Cpx response was inactivated, suggesting that the Cpx pathway assists protein turnover (Figures 4B,C). Given that bacteria with a compromised ETC are not able to generate sufficient amounts of ATP to support growth on non-fermentable carbon sources, we hypothesized that Cpx-regulated protein folding and degrading factors may impact the biogenesis of the ETC, together with the ability of the bacterial cells to generate proton motive force (PMF) and maintain their ATP levels. The candidate gene list for this experiment was derived from previous publications (Price and Raivio, 2009; Raivio et al., 2013) and unpublished RNASeq data. To identify genes of interest, we performed a preliminary screening of several of these envelope-associated protein folding and degrading factors (unpublished data). Cpx-regulated genes were overexpressed in a Δ*nuoA* strain containing the NuoA-3 × FLAG expression vector and NuoA-3 × FLAG protein levels were analyzed by dot blot. Overexpression of the DegP, HtpX, PpiD, and YccA proteins had the largest impact on the abundance of NuoA-3 × FLAG protein, surprisingly resulting in a greater than 2-fold increase in the amount of NuoA-3 × FLAG in comparison to the vector control. Furthermore, the protease/chaperone DegP, the IM protease HtpX, proteolytic modulating factor YccA, and periplasmic chaperone PpiD, have all been implicated in maintaining the integrity of the envelope and responding to stress generated by protein misfolding (Danese et al., 1995; Shimohata et al., 2002; Yamamoto and Ishihama, 2006; Matern et al., 2010; Raivio et al., 2013).

**FIGURE 5:**
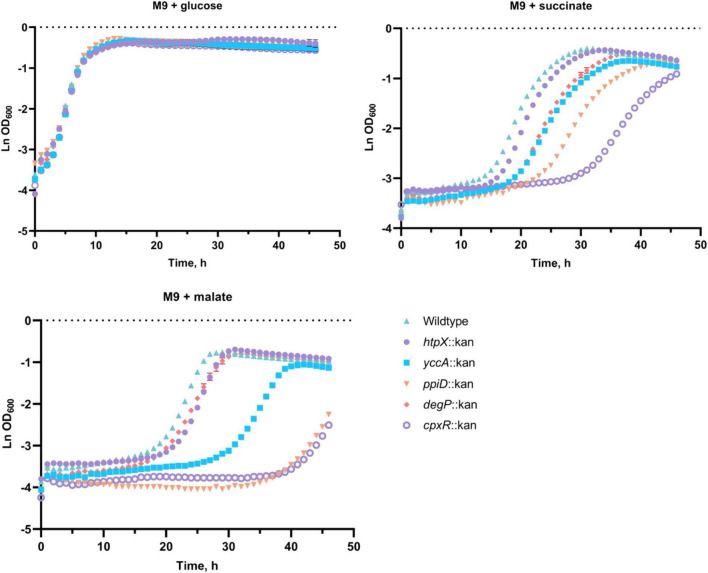
Deletion of several Cpx-regulated protein-folding and degrading factors results in growth defects in minimal media. Wildtype BW25113 and the mutants carrying the indicated gene deletions were grown overnight in LB at 37°C, washed twice in 1x phosphate-buffered saline (PBS), and standardized to an OD_600_ of 1.0 in phosphate buffered saline. 10 μL of the culture was subcultured into M9 minimal medium containing 0.4% glucose, 0.4% malic acid, pH 7.0 (malate) or 0.4% succinic acid, pH 7.0 (succinate), and grown for 48 h at 37°C with 330 rpm linear shaking. Data correspond to the mean values from three biological replicates and are representative of three independent experiments. Error bars depict standard deviations (SDs).

To investigate the role these proteins may have in the quality control of the ETC complexes, we analyzed growth of their knockout mutants in minimal media supplemented with glucose, succinate or malate in BW25113 *E*. *coli* (Figure 5; Kitagawa et al., 2005). As shown in Figure 5, deleting *cpxR*, *ppiD*, *yccA*, *htpX*, or *degP* resulted in growth defects of differing severity in comparison to the wildtype. All strains were able to grow on media supplemented with glucose, where their growth would not solely depend on energy generated via respiration. In addition, we noticed that the growth phenotypes of some mutants differed depending on whether the media contained malate or succinate as a carbon source.

Our data demonstrate that knocking out *cpxR*, *ppiD*, or *yccA* severely attenuates growth in media supplemented with malate, whereas these defects are less pronounced in succinate. Notably, deletion of genes encoding IM protease HtpX and periplasmic protease/chaperone DegP resulted in more modest growth defects (Figure 5). This result was somewhat surprising, as Cpx-mediated regulation of these proteolytic factors is an important element of envelope quality control (Jones et al., 2002; Shimohata et al., 2002), although it is possible their functions overlap with other proteolytic factors. To further demonstrate that these factors are required for respiratory growth, we performed a complementation assay where the mutants were transformed with an empty vector or a plasmid expressing the gene of interest. We were partially able to complement growth defects of every mutant in media where succinate or malate were the sole carbon source (Supplementary Figure 3). Together, these data demonstrate that the absence of a functional Cpx system and several associated protein folding and degrading factors compromises proper biogenesis of the ETC, resulting in decreased viability in media that demand respiration.

### Cpx-Regulated Protein Folding and Degrading Factors Affect NuoA Protein Levels

**FIGURE 6:**
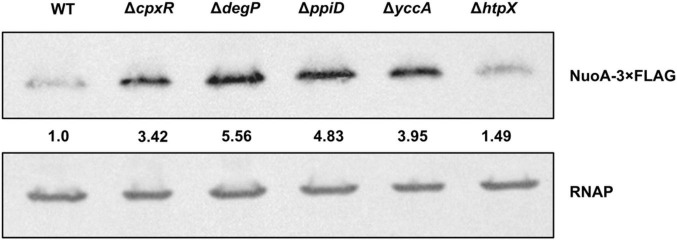
NuoA-3 × FLAG protein levels are altered by deletion of several Cpx-regulated protein folding and degrading factors. Western blot utilizing anti-FLAG tag antibody was performed on wildtype BW25113 *E*. *coli* and the mutants with the indicated gene deletions containing the pMPM-*nuoA*-3 × FLAG expression vector. RNAP protein levels served as a loading control. Membranes were imaged using the Bio-Rad ChemiDoc MP imaging system. Relative band quantification was performed using band intensity analysis in ImageJ. Band intensity is represented by the value relative to the wildtype NuoA-3 × FLAG protein level.

Our results implicate the Cpx-regulated envelope quality control factors YccA and PpiD, and to a lesser extent the proteases DegP and HtpX, in the biogenesis of the ETC (Figures 4, 5). We therefore hypothesized that knocking out *ppiD*, *yccA*, *degP*, or *htpX* may impact NuoA-3 × FLAG protein abundance. Here, we demonstrate that deletion of these factors alters NuoA-3 × FLAG protein levels compared to the wildtype (Figure 6). Deletion of *degP*, *ppiD*, *yccA*, *or htpX* increased NuoA-3 × FLAG abundance by a factor of 5.56, 4.83, 3.95, and 1.49, respectively, in comparison to wildtype. Although the specific fold changes in NuoA-3 × FLAG levels varied between biological replicates within this experiment, we consistently detected an increased amount in the mutant strains relative to the wildtype. In agreement with our previous findings, deletion of CpxR also resulted in accumulation of NuoA-3 × FLAG (Figures 4A, 6). Overall, our results suggest that several Cpx-regulated protein folding and degrading factors affect abundance of the NuoA protein and support the hypothesis of the Cpx-dependent regulation of the NDH-I complex at the post-translational level.

## Discussion

The Cpx response mediates adaptation to stresses that result in envelope perturbations through a variety of mechanisms, including facilitating degradation of misfolded or mislocalized proteins. Until recently, there was only a small number of proteins whose stability was known to be affected by the Cpx stress response; the breadth of known Cpx-mediated adaptations, however, continues to expand. It was recently demonstrated that the Cpx response regulates the expression of genes encoding NDH-I and cytochrome *bo*_3_, large envelope complexes involved in aerobic respiration (Guest et al., 2017). Transcriptional repression of the *nuo* and *cyo* promoter regions under stress is thought to reduce production of new respiratory complexes and prevent excessive protein traffic within an already compromised cell envelope (Guest et al., 2017). Interestingly, when the rates of oxygen consumption were compared between Δ*cpxRA* and wildtype EPEC, it was found that despite increased transcription of the *nuo* and *cyo* operons in the Δ*cpxRA* mutant, the activity of the aerobic ETC was still impaired (Guest et al., 2017). These discoveries suggested a novel role for the Cpx TCS in monitoring protein biogenesis and regulating factors, potentially impacting the function, stability, and assembly of respiratory complexes beyond transcription.

In this study, we provide evidence that the Cpx response regulates succinate dehydrogenase, the only enzyme of the TCA cycle that interacts directly with the ETC chain, being central to cellular metabolism and energy conversion (Hederstedt and Rutberg, 1981; Cecchini et al., 2002; Yankovskaya et al., 2003; Price and Driessen, 2010; Kaila and Wikström, 2021). Raivio et al. (2013) reported that the *sdhCDAB* operon was among the genes whose transcription was downregulated upon transient NlpE overexpression, along with other genes involved in respiration, including the *nuo* and *cyo* operons. In agreement with this finding, we show that the Cpx TCS inhibits the activity of a p*sdhC*::*lux* luminescent reporter in a wildtype strain under standard laboratory conditions, whereas in the absence of a functional Cpx response, *sdhC* promoter expression is elevated (Figures 1C,D).

How might this operon be regulated by the Cpx response? Previous studies have shown that many Cpx-regulated genes contain a CpxR binding site within 100 bp of their transcriptional start site (Price and Raivio, 2009). Given that both the *nuo* and *cyo* promoter regions possess CpxR binding sites, and their transcription is mediated though direct binding of CpxR, it is possible *sdhCDAB* is regulated similarly. We have identified a putative CpxR binding site approximately 150 bp downstream of the *sdhCDAB* TSS by using Virtual Footprint software (Münch et al., 2005; Guest et al., 2017). However, further experimentation is required to confirm this mechanism. Binding of the CpxR response regulator between the TSS and the translation start site would block transcription elongation from the *sdhC* promoter, decreasing the overall rate of *sdhCDAB* transcription. It is important to note though that this site deviates from the CpxR consensus binding sequence [5′-GTAAA(N_5_)GTAAA-3′], possessing a 6 bp linker and containing a GTTAA sequence in the 5′ half of the binding site (Supplementary Figure 1; De Wulf et al., 2002). Interestingly, several other transcriptional regulators also have binding sites near the *sdhCDAB* TSS; there are four ArcA binding motifs (Park et al., 1995; Shen and Gunsalus, 1997) and one for the cyclic adenosine monophosphate (cAMP)-cAMP receptor protein (CRP) complex, which is located approximately 94 bp upstream of the *sdhC* TSS (Zheng et al., 2004; Surmann et al., 2020). Other known regulators of the *sdhCDAB* operon include Fur (Zhang et al., 2005; Kumar and Shimizu, 2011), Fnr (Park et al., 1995) and several sRNAs shown to inhibit *sdhCDAB* mRNA translation (Massé and Gottesman, 2002; Geissmann and Touati, 2004; Desnoyers and Massé, 2012). It is possible that some of these regulators may work in combination with CpxR or other members of the Cpx TCS to regulate the transcription and/or translation of the *sdhCDAB* operon in response to environmental changes.

Our results indicate that the wildtype activity of the Cpx pathway leads to transcriptional repression of the *sdhCDAB* gene cluster. To validate our findings, and given that oxygen consumption in EPEC was shown to be affected by excessive activation of the Cpx response (Guest et al., 2017), we examined the rates of succinate oxidation by SDH in different Cpx backgrounds (Figure 2). We found that both NlpE overexpression and constitutive activation of the Cpx response by the *cpxA24* allele resulted in decreased SDH activity, which is consistent with a decrease in *sdhCDAB* expression under these conditions (Figure 2; Raivio et al., 2013). Unexpectedly, we observed similarly low enzymatic activity in a mutant lacking the Cpx pathway, which correlated with the reduced ETC performance in Δ*cpxRA* mutant relative to the wildtype strain demonstrated previously (Guest et al., 2017).

Evidently, both excessive activation and the absence of the Cpx stress response impacts the SDH complex, directly regulating transcription of the *sdhCDAB* operon, and potentially influencing the biogenesis of the complex. Given the role of the Cpx TCS in detecting and responding to potentially lethal misfolded proteins at the IM, it has been hypothesized that its housekeeping activity contributes to proper folding, stability and regulated turnover of large envelope complexes (Hung et al., 2001; Price and Raivio, 2009; Raivio et al., 2013; Hews et al., 2019). While a significant portion of the Cpx regulon consists of proteases and chaperones that address protein misfolding at the IM, to our knowledge, there are no known proteolytic factors involved specifically in degradation or stability of the ETC. Nevertheless, general polypeptide misfolding can be recognized by other non-specific proteases, including HtpX, DegP, and FtsH, which are known responders to envelope stress and are either directly or indirectly regulated by the Cpx pathway (Kihara et al., 1995; Akiyama et al., 1996; Jones et al., 2002; Shimohata et al., 2002; Isaac et al., 2005; Sakoh et al., 2005; van Bloois et al., 2008). The absence of such quality control in a Δ*cpxRA* knockout mutant could lead to aberrant complex formation and unproductive interactions between subunits of the respiratory complexes, leading to loss of function.

We found that the increased demand for respiratory complexes resulting from utilization of non-fermentable carbon sources acts as an inducing cue for the Cpx response (Figure 3; Bongaerts et al., 1995). Envelope stress can be exacerbated by the elevated activity of respiratory complexes. However, in *E*. *coli* this stress is not likely to be due to disruption of the proton gradient; the Cpx response is not induced by the chemical protonophore carbonyl cyanide 3-chlorophenylhydrazone (CCCP) (Engl et al., 2011; Guest et al., 2017). It is possible that in order to generate sufficient energy from non-fermentable carbon sources, bacterial cells upregulate respiratory complexes, which can then be subject to misassembly, irreparable damage or insertion into an already compromised membrane, a situation that would require a functional Cpx envelope stress response. Previous studies demonstrated that bacteria carrying mutations in genes encoding components of the ETC and quinone biosynthesis exhibit growth defects on succinate, malate, lactate, and acetate (Au and Gennis, 1987; Prüss et al., 1994; Kervinen et al., 2004; McNeil et al., 2012; Aussel et al., 2014). We observe similar growth phenotypes in the absence of the Cpx response, or Cpx-regulated protein folding and degrading factors (Figures 3, 5).

Upregulation of envelope quality control factors constitutes a part of Cpx-mediated adaptation under conditions in which damaged and/or misfolded proteins are predicted to accumulate (Hews et al., 2019; Mitchell and Silhavy, 2019). Regulation of proteolysis at the IM of *E*. *coli* is one of the least characterized parts of the Cpx response and is essential for maintaining the integrity of envelope biogenesis (Akiyama, 2009; Raivio, 2018; Delhaye et al., 2019a; Hews et al., 2019). Here, we demonstrate that the NuoA subunit of the NDH-I complex is subject to regulation by the Cpx pathway beyond transcription, and that activation of the Cpx response decreases the abundance of NuoA-3 × FLAG protein (Figure 4A). Furthermore, we show that the efficiency of NuoA-3 × FLAG protein turnover is reduced in the absence of the functional Cpx response (Figures 4B,C). These results can be interpreted in two ways. First, the Cpx system may regulate factors that inhibit translation, such as the CpxQ sRNA (Chao and Vogel, 2016), therefore decreasing the amount of successfully translated *nuoA*-3 × FLAG mRNA. Interestingly, CpxQ has been previously proposed to play a role in preserving the PMF at the IM since it downregulates the sodium-proton antiporter NhaB and counteracts the loss of membrane potential caused by CCCP treatment (Chao and Vogel, 2016). Moreover, several other sRNAs have been implicated in the post-transcriptional regulation of the ETC complexes, including RyhB that regulates both *nuo* and *sdhCDAB* transcripts under low-iron conditions (Massé and Gottesman, 2002; Massé et al., 2005; Desnoyers and Massé, 2012).

Additionally, activation of the Cpx response may result in increased proteolysis of existing NuoA-3 × FLAG proteins. This model is supported by the fact that the Cpx response regulates the expression of proteolytic factors responsible for quality control at the IM (Raivio et al., 1999, 2013; Shimohata et al., 2002; Price and Raivio, 2009; Raivio, 2014). Our data demonstrates that loss of these factors results in growth defects in media that requires a functional ETC for survival, which is in agreement with the previously suggested role of the Cpx response in the biogenesis of respiratory proteins (Figure 5; Guest et al., 2017). Together, these findings strengthen the link between the ETC and the Cpx TCS, where in addition to direct transcriptional repression of respiratory complexes, the Cpx pathway regulates their biogenesis and turnover, likely through the controlled expression of protein folding and degrading factors.

Considering the impact the Cpx response has on aerobic respiration, why does the availability of relatively similar non-fermentable carbon sources in the medium yield such different growth outcomes (Figure 5)? Aside from the ETC biogenesis, the other key factor in the minimal media experiments is the differential energetics and interactions of the non-fermentable carbon sources with bacterial central catabolism. Despite being fed directly into the TCA cycle, malate and succinate differentially contribute to ETC bioenergetics. Malate and its oxidation product NADH are stronger reducing agents, with an E′_0_ of –0.166 and –0.320 V, respectively, compared to succinate and its oxidation product FADH_2_ with an identical E’_0_ of +0.031 V (Karp, 2008). In other words, the oxidation of malate resulting in NADH production and subsequent oxidation of NADH by NDH-I provides the cell more energy for ATP production. If NDH-I is functionally deficient, NADH-derived electrons cannot be utilized directly for proton motive force generation, given that the NDH-II complex is not involved in proton translocation (Matsushita et al., 1987; Minárik et al., 2002) and ATP generation is dependent on the proton-pumping activity of the downstream cytochromes. This is supported by the fact that the inhibition of NADH oxidase subunit activity of NDH-I results in poor to no growth on malate (Kao et al., 2005). Therefore, removing factors potentially involved in the biogenesis of NDH-I may result in strong growth defects when malate is the sole carbon source. It is possible that the ability to bypass NDH-I and metabolize succinate directly through succinate dehydrogenase results in better viability of these mutants.

Here, we propose that several envelope quality control factors, some of which are Cpx-regulated, affect the biogenesis of NDH-I and, potentially, other respiratory complexes. The inability to utilize non-fermentable carbon sources (Figure 5) by the strains lacking envelope quality control could be caused by lethal accumulation of irreparably damaged respiratory complexes in the absence of appropriate protein turnover (Figures 4B,C, 6). While we did not determine whether YccA, DegP, PpiD, or HtpX impact the ETC complexes directly, we know that most of these are associated with the non-specific protease FtsH and SecYEG-dependent translocation (Kihara and Ito, 1998; Antonoaea et al., 2008; Van Stelten et al., 2009; Leiser et al., 2012; Fürst et al., 2018). FtsH-mediated degradation of the SecY subunit of the SecYEG translocon prevents blocking of the translocase with inefficiently exported proteins (Langklotz et al., 2012).

Interestingly, not all mutations were similarly detrimental to the cell. For instance, *htpX* mutants displayed relatively mild growth defects (Figure 5) and reductions in NuoA-3 × FLAG protein levels (Figure 6), possibly because HtpX functions as a protease with cellular roles complementary or overlapping to those of FtsH (Leiser et al., 2012). In contrast, removing proteins involved in modulation of FtsH proteolytic activity, including YccA and DegP, could lead to excessive degradation of SecY, not allowing for damaged IM proteins to be replaced. PpiD, on the other hand, may assist with secretion and folding of membrane parts of the NDH-I complex. One of the proposed mechanisms of NDH-I assembly involves its co-translational translocation through the Sec translocon and further insertion of its integral protein into the membrane (Friedrich et al., 2016); however, the chaperones involved in this process are yet to be described. PpiD has been previously shown to improve translocation efficiency by clearing the Sec translocon of newly synthesized proteins (Fürst et al., 2018), while its deletion leads to delayed release of proteins from the cytoplasmic side (Antonoaea et al., 2008).

Cumulatively, our results suggest that activation of the Cpx response may stimulate FtsH proteolytic degradation and/or impact the secretion of the electron transport chain proteins. However, since *ftsH* is essential in *E*. *coli* (Akiyama et al., 1994), we were unable to determine if it is required for Cpx-mediated degradation of NuoA-3 × FLAG. A major future direction could involve deleting *ftsH* in a strain that carries the *sfhC21* allele, which suppresses the lethality of the *ftsH* null and assessing NuoA-3 × FLAG levels (Ogura et al., 1999).

**FIGURE 7:**
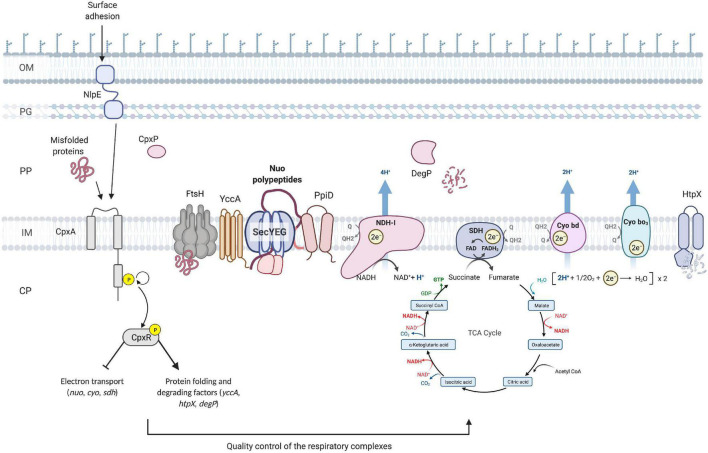
Model of the quality control of the ETC performed by the Cpx envelope stress response at the inner membrane of *E*. *coli*. Upon activation of the Cpx response, CpxR represses the transcription of the operons encoding the NDH-I (*nuo*), cytochrome *bo*_3_ (*cyo*) and succinate dehydrogenase (*sdh*) respiratory complexes. In addition, the Cpx response regulates the biogenesis of these complexes beyond transcription through increased expression of protein folding and degrading factors. Cpx-regulated protein folding factors assist with the secretion and further insertion of integral parts of the ETC complexes, whereas the proteolytic factors maintain the adequate turnover and degradation of the misfolded or mislocalized proteins. OM, outer membrane; PG, peptidoglycan; PP, periplasm; CP, cytoplasm; Q, quinone; Cyo, cytochrome.

One of the major findings of this work is that another essential component of cellular energetics, the succinate dehydrogenase complex, is a member of the Cpx regulon and that its expression is downregulated in response to stresses sensed by the Cpx TCS. Furthermore, the housekeeping activity of the Cpx response is required for proper biogenesis and performance of succinate dehydrogenase, as evidenced by the fact that SDH activity is impaired in the absence of the functional Cpx pathway. Recent studies hypothesize that during normal biogenesis of the ETC complexes, some subunits may not assemble correctly, and these subunits engage in non-productive interactions that generate the stress sensed by the Cpx response (Guest et al., 2017). This hypothesis is further supported by our findings, where increased demand for respiratory complexes induces the Cpx pathway, possibly due to a higher risk of protein misfolding. Subsequently, activation of the Cpx response results in upregulation of proteases that degrade existing complexes, possibly facilitate secretion and membrane insertion, and directly repress *nuo*, *sdhCDAB* and *cyo* transcription. Altogether, our results support a model in which the Cpx pathway maintains the function of critical cytoplasmic membrane protein complexes through modulation of an intricate balance between transcriptional repression and increased protein turnover during periods of stress, while allowing for recovery of vital cellular activities including translocation of newly synthesized proteins and their insertion into the membrane as envelope stress is alleviated (Figure 7).

## Data Availability Statement

All datasets generated for this study are included in the article/Supplementary Material.

## Author Contributions

VT was involved in the design of the study, the acquisition, analysis, and interpretation of the data, and wrote the manuscript. RG was involved in the design of the study, the acquisition, analysis, and interpretation of the data. TR was involved in the conception and design of the study, the interpretation of the data, as well as wrote the manuscript. All authors contributed to the article and approved the submitted version.

## Conflict of Interest

The authors declare that the research was conducted in the absence of any commercial or financial relationships that could be construed as a potential conflict of interest.

## Publisher’s Note

All claims expressed in this article are solely those of the authors and do not necessarily represent those of their affiliated organizations, or those of the publisher, the editors and the reviewers. Any product that may be evaluated in this article, or claim that may be made by its manufacturer, is not guaranteed or endorsed by the publisher.

## Footnotes

1
https://www.prodoric.de/vfp
