# Supplementary material for: The Cpx Stress Response Regulates Turnover of Respiratory Chain Proteins at the Inner Membrane of Escherichia coli
Source: Front Microbiol. 2022 Jan 28;12:732288. doi: 10.3389/fmicb.2021.732288 (PMC8831704; doi:10.3389/fmicb.2021.732288)
Supplement: Supplementary file 1 [file Data_Sheet_1.docx]

Supplementary Material

**Materials and Methods**

**Complementation assay.**

Wildtype BW25113 or knockout mutants containing either a vector control plasmid pCA-24N or the plasmid expressing gene of interest were grown overnight in 2mL LB containing appropriate concentrations of antibiotic at 37°C with shaking. The following day, cells were pelleted by centrifugation and washed twice with 1×PBS. The cell density was standardized to OD_600_ 1.0 by suspending an appropriate volume of cells in 1 ml of 1×PBS. 20µL of each sample was loaded into a 96-well plate, each well containing 180µL of either 0.4% glucose (Sigma), 0.4% malic acid, pH 7.0 (Sigma) or 0.4% succinic acid, pH 7.0 (Sigma) M9 minimal medium (Difco) containing appropriate concentrations of antibiotics. Leaky expression from the plasmids was used in this experiment. The plate was incubated in the Agilent BioTek Epoch 2 Microplate Spectrophotometer with 330 rpm shaking at 37℃ for 48 hours.

**Tables and Figures**

**
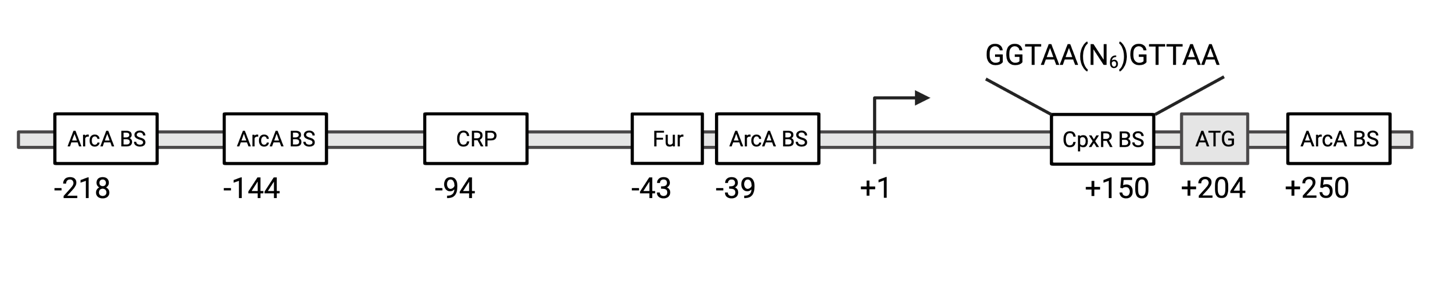
**

**Figure S1. Identification of a putative CpxR binding site upstream of *sdhC*.** Schematic representation of the *sdhC* promoter region of BW25113 *E. coli* indicating the locations of the putative CpxR, ArcA, Fur and CRP-cAMP binding sites. Numbers indicate distances of the most upstream bp in each site from the transcription start site (+1) in base pairs. -, upstream; +, downstream; BS, binding site.

**
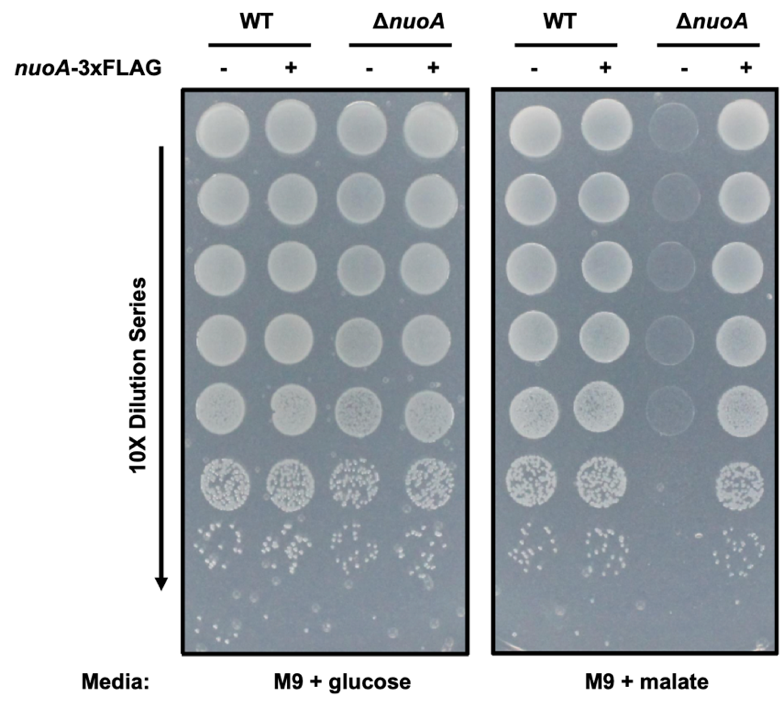
**

**Figure S2. NuoA-3xFLAG construct is functional.** Wildtype BW25113 and the Δ*nuoA* mutant containing either the empty pMPM-K3 vector (-) or pMPM-*nuoA*-3xFLAG (+) were grown overnight in LB medium, washed once in phosphate-buffered saline, and diluted to an OD_600_ of 1.0 in phosphate buffered saline (PBS). 10μL of 10-fold serial dilutions were spotted onto minimal medium (MM) agar containing 0.4% malic acid, pH 7.0 (malate) or 0.4% glucose and bacteria were grown overnight at 37℃.


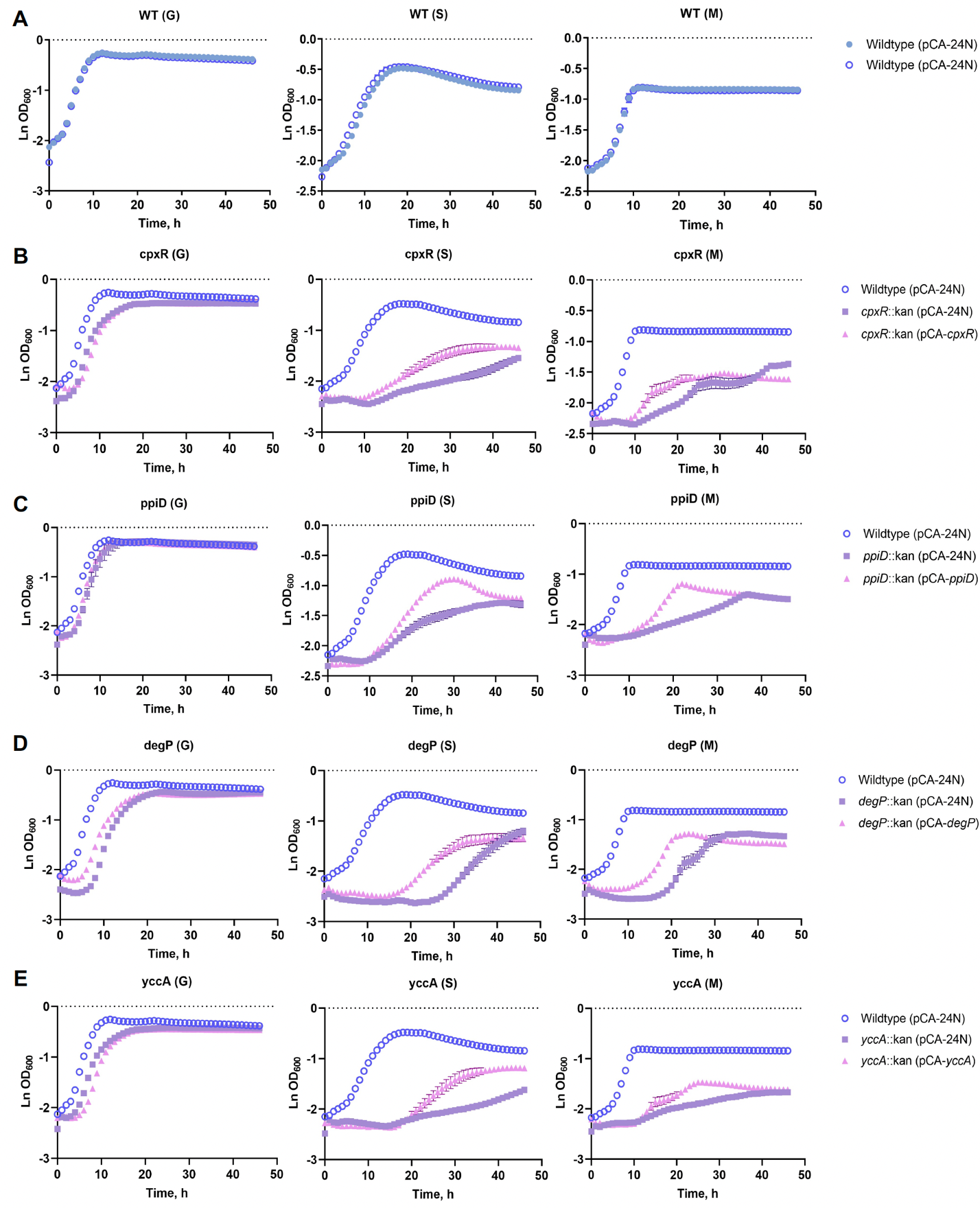


**Figure S3. Exogenous expression of several Cpx-regulated protein-folding and degrading factors partially restores growth defects of mutants in minimal media.** Wildtype BW25113 and the mutants carrying the indicated gene deletions containing either a vector control plasmid pCA-24N or the plasmid expressing gene of interest were grown for 48 hr at 37°C with 330 rpm linear shaking in M9 minimal medium containing 0.4% glucose (G), 0.4% malic acid, pH 7.0 (M) or 0.4% succinic acid, pH 7.0 (S). Data correspond to the mean values from three biological replicates and are representative of two independent experiments. Error bars depict standard deviations (SDs).

**Table S1. Bacterial strains and plasmids used in this study**

| Strain or plasmid | Description | Source or reference | |
| --- | --- | --- | --- |
| *Bacterial strains* | | | |
| MC4100 | F^-^, *[araD139]_B/r_, Δ(argF-lac)169, λ^-^, e14-, flhD5301, Δ(fruK-yeiR)725(fruA25), relA1, rpsL150(*StrR*), rbsR22, Δ(fimB-fimE)632(::IS1), deoC1* | (Casadaban, 1976) |  |
| VT2 | MC4100 *cpxR*::kan | This study |  |
| E2348/69 | Prototypical EPEC O127:H6 laboratory strain | (Levine et al., 1978) |  |
| RG222 | E2348/69 *ΔcpxRA* | (Guest et al., 2017) |  |
| ALN195 | E2348/69 *cpxA24* | (Macritchie et al., 2008) |  |
| BW25113 | F^-^, *Δ(araD-araB)567, lacI^+^, ΔlacZ4787(*::rrnB-3*), λ^-^, rph-1, Δ(rhaD-rhaB)568, hsdR514* | (Datsenko and Wanner, 2000; Baba et al., 2006) |  |
| TR10 | MC4100 *cpxA24* | (Raivio and Silhavy, 1997) |  |
| JW1818 | BW25113 *htpX::*kan | (Baba et al., 2006) | |
| JW0953 | BW25113 *yccA::*kan | (Baba et al., 2006) | |
| JW0431 | BW25113 *ppiD::*kan | (Baba et al., 2006) | |
| JW0157 | BW25113 *degP::*kan | (Baba et al., 2006) | |
| JW3883 | BW25113 *cpxR*::kan | (Baba et al., 2006) | |
| ﻿JW2283 | ﻿BW25113 *nuoA*::kan | (Baba et al., 2006) | |
| RG349 | E2348/69 (pMPM-*nuoA*-3**×**FLAG) | This study | |
| RG351 | E2348/69 *cpxA24* (pMPM-*nuoA*-3**×**FLAG) | This study | |
| RG350 | E2348/69 *cpxR*::spc (pMPM-*nuoA*-3**×**FLAG) | This study | |
| VT115 | MC4100 (p*cpxP::lux*) | This study | |
| VT123 | MC4100 (pJW15) | This study | |
| VT124 | MC4100 (p*sdhC::lux*) | This study | |
| RG57 | MC4100 (p*nuoA::lux*) | (Guest et al., 2017) | |
| VT130 | MC4100 *cpxA*::cam (p*cpxP::lux*) | This study | |
| VT131 | MC4100 *cpxA*::cam (p*sdhC::lux*) | This study | |
| VT132 | MC4100 *cpxA*::cam (pJW15) | This study | |
| VT133 | MC4100 *cpxA*::cam (p*nuoA::lux*) | This study | |
| TC220 | MC4100 (pTrc-*nlpE*) | This study | |
| VT179 | BW25113 *cpxR*::kan (pCA-*cpxR*) | This study | |
| VT180 | BW25113 *cpxR*::kan (pCA-24N) | This study | |
| VT183 | BW25113 *degP*::kan (pCA-*degP*) | This study | |
| VT184 | BW25113 *degP*::kan (pCA-24N) | This study | |
| VT185 | BW25113 *yccA*::kan (pCA-*yccA*) | This study | |
| VT186 | BW25113 *yccA*::kan (pCA-24N) | This study | |
| VT187 | BW25113 *ppiD*::kan (pCA-*ppiD*) | This study | |
| VT188 | BW25113 *ppiD*::kan (pCA-24N) | This study | |
| RG480 | BW25113 Δ*nuoA* derivative of JW2283 | (Guest et al., 2017) | |
| *Plasmids* | | | |
| pFLP2 | Broad host-range plasmid expressing the FLP recombinase from a temperature sensitive promote; Amp^R^ | (Eperiments with Gene Fusions, 1985) | |
| pJW15 | Luminescence reporter vector plasmid | (Wong et al., 2013) | |
| pJW15-p*cpxP* (pJW25) | Luminescence reporter plasmid containing *cpxP* promoter; Kan^R^ | (Macritchie et al., 2008) | |
| pJW15-p*nuoA* | Luminescence reporter plasmid containing *nuoA* promoter; Kan^R^ | (Guest et al., 2017) | |
| pJW15-p*sdhC* | Luminescence reporter plasmid containing *sdhC* promoter; Kan^R^ | (Guest et al., 2017) | |
| pMPM-K3 | Low copy-number IPTG inducible cloning vector derived from pACYC184 and pBluescript; Kan^R^ | (Mayer, 1995) | |
| pMPM-NuoA-3×FLAG | pMPM-K3 derived IPTG inducible *nuoA*-3xFLAG expression vector; Kan^R^ | (Guest, 2017) | |
| pTrc-*nlpE* | High copy-number expression vector with IPTG inducible *nlpE* promoter derived from pTrc99A vector; Amp^R^ | This study | |
| pCA-24N | Vector control for ASKA library containing the P_T5-lac_ IPTG-inducible promoter; Cam^R^ | (Kitagawa et al., 2005) | |
| pCA-*degP* | IPTG-inducible degP overexpression vector from the ASKA library; CamR | (Kitagawa et al., 2005) | |
| pCA-*ppiD* | IPTG-inducible *ppiD* overexpression vector from the ASKA library; Cam^R^ | (Kitagawa et al., 2005) | |
| pCA-*cpxR* | IPTG-inducible *cpxR* overexpression vector from the ASKA library; Cam^R^ | (Kitagawa et al., 2005) | |
| pCA-*yccA* | IPTG-inducible *yccA* overexpression vector from the ASKA library; Cam^R^ | (Kitagawa et al., 2005) | |

**Table S2. Oligonucleotide primers used in this study**

| Primer name | Sequence |
| --- | --- |
| nuoAFLAGFwd | 5’-TTTTAAGCTTCTTTTGATGAGTA-3’ |
| nuoAFLAGRev | 5’-TTTTTCTAGATTATTTATCATCATCATCTTTATAATCAATATCATG ATCTTTATAATCGCCATCATGATCTTTATAATCGCGTTGACGATTAG CGATAC-3’ |
| PsdhCFwdCln | 5’- TTTTGAATTCGGTCTACCACTAATAACTG-3’ |
| PsdhCRevCln | 5’- TTTTGGATCCATGGAGAATGGACGCTATC-3’ |
| K1 | 5’-CAGTCATAGCCGAATAGCCT-3’ |
| M13F | 5’-GTTTTCCCAGTCACGAC-3’ |
| M13R | 5’-AACAGCTATGACCATG-3’ |
| nlpE_NcoI_F | 5’-CGCACCATGGTGAAAAAAGCGATAGTGACAG-3’ |
| nlpE_WT_His_HindIII_R | 5’-TGCCAAGCTTTTAGTGGTGGTGGTGGTGGTGCTCGAGCTGCCCCAAACTACTGCAATC-3’ |
| pTrc99A_F | 5’-GTTCTGGCAAATATTCTGAAA-3’ |
| pTrc99A_R | 5’-ATTTAATCTGTATCAGGCTGA-3’ |
| P1cpxRFwd | 5’-AACTATGCGCATCATTTGCTCC-3’ |
| P1cpxR Rev | 5’-CACATTAAATCGTTGGGCGGAT-3’ |

*Underlined sequences indicate restriction endonuclease cut sites (HindIII: AAGCTT, XbaI: TCTAGA)

**P1 transduction strains were verified by PCR using gene-specific forward primers (P1*genename*Fwd) and K1 reverse primer that binds inside of the kanamycin resistance gene.

**Table S3. Relative band quantification calculated using Fiji (ImageJ) software for Figure 4B.**

| Time, min | BW25113 WT | BW25113 *cpxR*::spc | BW25113 *cpxA24* |
| --- | --- | --- | --- |
| 0 | 1 | 1 | 1 |
| 1 | 0.8736227 | 0.83863009 | 0.96293758 |
| 5 | 0.71124593 | 0.75016512 | 0.72735328 |
| 10 | 0.63119508 | 0.70426201 | 0.76737539 |
| 20 | **0.49823794** | 0.64889856 | **0.44952824** |
| 30 | 0.44765134 | 0.64664517 | 0.4140362 |
| 45 | 0.35571218 | 0**.47736897** | 0.24964914 |
| 90 | 0.17522416 | 0.47630056 | 0.12982889 |
| 120 | 0.15862961 | 0.52593341 | 0.11517807 |

*Values represent relative quantification of the raw integrated density of the protein bands.

**The closest value representative of the protein half-life is highlighted in bold.

**BIBLIOGRAPHY**

Baba, T., Ara, T., Hasegawa, M., Takai, Y., Okumura, Y., Baba, M., et al. (2006). Construction of Escherichia coli K-12 in-frame, single-gene knockout mutants: The Keio collection. *Mol. Syst. Biol.* 2. doi:10.1038/msb4100050.

Casadaban, M. J. (1976). Transposition and fusion of the lac genes to selected promoters in Escherichia coli using bacteriophage lambda and Mu. *J. Mol. Biol.* 104, 541–555. doi:10.1016/0022-2836(76)90119-4.

Datsenko, K. A., and Wanner, B. L. (2000). One-step inactivation of chromosomal genes in Escherichia coli K-12 using PCR products. *Proc. Natl. Acad. Sci. U. S. A.* 97, 6640–6645. doi:10.1073/pnas.120163297.

Eperiments with Gene Fusions (1985). *J. Basic Microbiol.* 25, 350. doi:https://doi.org/10.1002/jobm.3620250516.

Guest, R. L. (2017). Regulation of respiration by the Cpx response in enteropathogenic Escherichia coli. [PhD thesis]. [Edmonton (AB)]: University of Alberta

Guest, R. L., Wang, J., Wong, J. L., and Raivio, T. L. (2017). A Bacterial Stress Response Regulates Respiratory Protein Complexes To Control Envelope Stress Adaptation. 199, 1–14. doi:10.1128/JB .00153-17.

Kitagawa, M., Ara, T., Arifuzzaman, M., Ioka-Nakamichi, T., Inamoto, E., Toyonaga, H., et al. (2005). Complete set of ORF clones of Escherichia coli ASKA library (a complete set of E. coli K-12 ORF archive): unique resources for biological research. *DNA Res. an Int. J. rapid Publ. reports genes genomes* 12, 291–299. doi:10.1093/dnares/dsi012.

Levine, M. M., Bergquist, E. J., Nalin, D. R., Waterman, D. H., Hornick, R. B., Young, C. R., et al. (1978). Escherichia coli strains that cause diarrhoea but do not produce heat-labile or heat-stable enterotoxins and are non-invasive. *Lancet (London, England)* 1, 1119–1122. doi:10.1016/s0140-6736(78)90299-4.

Macritchie, D. M., Ward, J. D., Nevesinjac, A. Z., and Raivio, T. L. (2008). Activation of the Cpx envelope stress response down-regulates expression of several locus of enterocyte effacement-encoded genes in enteropathogenic Escherichia coli. *Infect. Immun.* 76, 1465–1475. doi:10.1128/IAI.01265-07.

Mayer, M. P. (1995). A new set of useful cloning and expression vectors derived from pBlueScript. *Gene* 163, 41–46. doi:10.1016/0378-1119(95)00389-n.

Raivio, T. L., and Silhavy, T. J. (1997). Transduction of envelope stress in Escherichia coli by the Cpx two-component system. *J. Bacteriol.* 179, 7724–7733. doi:10.1128/jb.179.24.7724-7733.1997.

Wong, J. L., Vogt, S. L., and Raivio, T. L. (2013). Using reporter genes and the Escherichia coli ASKA overexpression library in screens for regulators of the Gram negative envelope stress response. *Methods Mol. Biol.* 966, 337–357. doi:10.1007/978-1-62703-245-2_21.
